# Therapeutic benefits of green tea extract on various parameters in non-alcoholic fatty liver disease patients

**DOI:** 10.12669/pjms.334.12571

**Published:** 2017

**Authors:** Mazhar Hussain, Habib-Ur-Rehman, Lubna Akhtar

**Affiliations:** 1 Dr. Mazhar Hussain, MBBS, M.Phil (Pharmacology), Department of Pharmacology & TherapeuticsSheikh Zayed Medical College, Rahim Yar Khan, Punjab, Pakistan; 2 Dr. Habib-Ur-Rehman, MBBS, MD, FCPS (Medicine), Department of Medicine, Sheikh Zayed Medical College & Hospital, Rahim Yar Khan, Punjab, Pakistan; 3 Dr. Lubna Akhtar, MBBS, FCPS (Gynae&Obs), Department of Pharmacology & TherapeuticsSheikh Zayed Medical College, Rahim Yar Khan, Punjab, Pakistan

**Keywords:** Aminotransferases, Dyslipidemia, Green tea extract, Non alcoholic fatty liver disease

## Abstract

**Background and Objective:**

NAFLD affecting up to 30% of the population globally. Drug treatment options are limited with disappointing results. The dietary supplementation in the form of green tea is another option. Our objective was toinvestigate the effect of Green tea extract (GTE) supplementation on various parameters innon-alcoholicfatty liver disease (NAFLD) patients.

**Methods:**

This study was conducted Dept. of Medicineof Sheikh Zayed Medical College/Hospital, Rahim Yar Khan from 15 April 2016 to 15 July 2016. Eighty overweight, non diabeticand dyslipidemic patients of NAFLD, diagnosed on the basis of ultrasound and aminotransferases level were randomized for treatmentwith capsule GTE500mg (n=40)and capsule placebo (n=40) twice a day for twelve weeks. Anthropometric parameters, liver enzymes, inflammatory markers and liver ultrasound imaging were estimated by SPSS-16 pre and post treatment.

**Results:**

As compared to placebo, GTE caused a significant improvement in body weight (29.5±3.8 to 27.2±3.2 kg/m^2^ p=0.03), BMI (86±10.5 to 80±12.4 kg p=0.026), HOMA-IR(4.32±2.25 to 3.16± 1.6 p=0.0081) lipid profile (i.e. TC: L242.5±20.5 to 215.4±18.6 mg/dl p=0.005; TG: 175±22.6 to145±18 mg/dlp=0.003; LDL-C:155±12.5 to 140±16.7 mg/dl p=0.011; HDL-C: 36.8±6.7 to46.4±5.8 mg/dl p =0.001, Aminotransferases (i.e. ALT: 70.4±15.8to52.8±12.2 IU/L p=0.04; AST: 65.8±12.4 to 44.3± 8.5U/L p =0.002) and Inflammatory markers (hs-CRP: 3.14±0.58 to 2.18±0.32 p =0.023 Adiponectin: 8.46±1.02 to 10.55±3.42μg/ml p =0.003)GTE also caused a 67.5% regression of fatty liver changes on ultrasound as compared to placebo which is 25%only.

**Conclusion:**

GTEtherapy resulted in significant improvement in metabolic, chemical, inflammatory and radiological parameters of non-alcoholic fatty liver disease patients who were non-diabetic anddyslipidemic.

## INTRODUCTION

Non alcoholic fatty liver disease (NAFLD) is foremost causeof progressive liver disorder worldwide. It has strong association with obesity 41-61%, metabolic syndrome 30-56%, Type-2 diabetes 18-28%, dyslipidemia 52-83% andhypertension 15-46%. These risk factors promote NAFLD as a global heath issue in western countries with a prevalence rate ofabout 20-30%. In recent years, similar prevalence rate in the range of 15-30% has been found in Asian countries in general population due to strong influence of life style modification and urbanization pattern to its associated risk factors. There is an urgent need to reduce the enormous clinical and economic burden of NAFLD worldwide.^1^,^2^

It is somewhat difficult to diagnose NAFLD clinically because most of the patients remain asymptomatic for years until they develop cirrhosis. Some patients may complain of tiredness and right upper quadrant distress or pain due to stretching of hepatic capsule but majority of them are diagnosed on the basis of deranged liver chemistryor abnormal ultrasound imaging studiesdone during routine medical checkup due toassociated metabolic conditions.^3^ NAFLD is a benign disease but if it cannot controlled appropriately it may progress tonon alcoholicsteatohepatitis(NASH), hepatic fibrosis, cirrhosis of liver, cardiovascular disorders, kidney diseases and is predicted to become the most common indication of liver transplantation by 2030.^4^

The criteria for the diagnosis of NADLD are usually based upon three findings. There is no history of alcohol use, detection of hepatic steatosis on imaging or histology andabsence of secondary causes of hepatic disorders. Transabdominalultrasound is usually preferred in clinical practice as an initial imaging modality in the diagnosis of NAFLD as compared to others tests because of its acceptability, availability, affordability, reliability, cost effectivenessandnon invasiveness. Italso providesqualitative information such as brightness of liver parenchyma, obscure of vascular margin, hepatorenal contrast and deep attenuation. In addition it can be used as a diagnostic tool for population screening on a broad scale asit usually not needs a specialsetup with protocol.^5^

Life style modification in the form diet, weight loss and physical exercises is advocated as the first line of intervention in NAFLD patients.^6^ However so far there is no effectivelicensed drug treatment forNAFLD patients. Due to the presence of associated comorbidities in NAFLD patients, various drugs such as insulin sensitizers, anti oxidants, antidyslipidemic, hepatoprotective (silymarin) and miscellaneous agents like pentoxifylline, orlistatand incretin based therapies with varying results have been tried up till now.^7^

Green tea is an unfermented product of the leaves and bar of the plant (Camellia sinensis) and isone of the famous drinks all over the world especially in the region of South EastAsia. Green tea contains thousands of bioactive compounds out of which one third is contributed by polyphenols which are mostly flavonoids.^8^ Catechins are one of the mainflavonoids in green tea has recently attracted attention for its use in various diseases due to its anti-aging, anti-cancer, anti-parkinsonism, anti-stroke, anti diabetic, anti-caries and anti anti-bacterial, anti-diarrheal, anti-fibrotic, anti-inflammatory, anti-oxidative and anti-atherosclerotic properties.^9^

The green tea has a strong potential to reverse all those suspected mechanisms that has been suspected in the pathogenesis of NAFLD in various clinical and experimental studies. Green tea decrease hepaticsteatosis by reducing hepatic insulin resistance which is a key mechanism in its pathogenesis.^10^ Green tea has a strong potential toboost immune system, reduces the formation of reactive oxygen species (ROS), increases the activity of pro-oxidant enzyme such as glutathione peroxidase and superoxide dismutase through it antioxidant properties and finallygreen tea reduces various inflammatorychemokines and cytokines as inflammation and oxidative stress is the pathognomonic features in NAFLD related complication.^11^ In addition green tea has beneficial effect on all NAFLD associated medical conditions such as obesity, metabolic syndrome,^12^ hypertension and dyslipidemia^13^ in various clinical studies.

So in this placebo controlled trials we evaluate the effect ofgreen tea extract (GTE) on variousanthropometric, chemical, metabolic, inflammatory and radiological parametersin NAFLD patients who are non diabetic as well as dyslipidemicin order to see its independent effect in non diabetics.

## METHODS

This study was designed as a randomized placebo controlled trial between two parallel groups and was carried out at Out Patients Department of Medical Unit-II at Sheikh Zayed Medical College/Hospital, Rahim Yar Khan from 15 April 2016 to 15 July 2016. On the basis of presenting complaints such as vague upper abdominal discomfort, indigestion and fatigue, initially two hundred patients were scrutinized for derangedliver enzyme and lipid profiles. From which eighty subjectswere registered in this clinical trial on the basis of following criteria. ***Inclusion criteria*** were age 20-55 years, BMI ≥ 27, elevated aminotransferases (mild to moderate), Ultrasound with fattyliver grading 1, 2, 3.***Exclusion criteria:*** It included noalcohol and drug abuse, smokers, pregnancy, lactation, diabetes mellitus, hypothyroidism, billiary disease, autoimmune diseases, drug induced hepatitis, chronic kidney disease, history of any cardiac diseases and decompensated liver disease. Patients who had highly abnormal ultrasound and aminotransferases level were also screened for viral hepatitis, hemochromatosis, and alpha-1 antitrypsin deficiency and Wilson disease. A detailed history was taken about drugs which effect fatty liver such as antidyslipidemic agents, anti diabetic drugs, amiodarone, corticosteroids, antiviral agents, tetracycline antibiotics, methotrexate and hormonal therapy. Same centre was selected for all patients to determine the parameters to avoid human error.

Patients were randomly divided in to two groups which were based upon random numbers generated by computer for each subject. The first group was given cap GTE 500mg twice daily for a period of twelve weeks while second groupwhichserved as a control were given cap placebo with same color, size, packing and duration but it contained microcrystalline cellulose as an active ingredient. Patients were instructed to continue their previous eating habits during the study period. This study was approved by the ethical committee of Sheikh Zayed Medical College/Hospital, Rahim Yar Khan. The purpose of study was clearly explained to the patients before written informed consent was obtained.

Pure green tea leaves were obtained from national tea research institute (NTRI) Mansehra Pakistan. Leaves were dried and a standardized method was used for the quantification analysis of major green tea catechins and caffeine components (Table-I) by Anultra high performance liquid chromatography (UHPLC) with UV detection in Pakistan Council of Scientific & Industrial Research (PCSIR) laboratory, Lahore, Pakistan. The 500mggreen tea capsule was prepared from GTE in pharmacy department of Sheikh ZayedMedical College, Rahim Yar Khan. Capsule placebo was made in a similar manner but it contains cellulose as an active ingredient. Blood samples were collected after an overnight fasting from cubitalveinat the beginning and end of the study to analyze bloodsugar, serum insulin, Homeostasis Model Assessment -Insulin resistance (HOMA-IR) index, lipid profile, liver enzyme, hs-CRP and serum adiponectin. In order to exclude diabetic patient’s blood sugar was analyzed by glucose oxidase peroxidase method at start of study. Serum lipid profiles and liver enzymes were estimated by semi-automated clinical chemistry analyzer (Micro Lab300)usingspectrophotometeric principal. Insulin level was measured with an insulin kit using a cobas immunoassay analyzer. Homeostasis Model Assessment -Insulin resistance (HOMA-IR) index was computed by the following equation fasting Glucose (mmol/L) insulin (mU/L)/22.5. The hs-CRP was detected with Latex-enhanced immune turbidimetric method (Orion, Finland) and serum adiponectin hormone was analysed withenzyme linked immunosorbent assay (ELISA)with adipogen kit Korea.

**Table-I:** Constituents of GTE in 500 mg each capsule.

*Constituents*	*GTE (% weight)*	*Placebo(% weight)*
Catechins	0.690	0
Caffeine	2.275	-
Epicatechin Gallate (ECG)	2.647	-
Gallocatechin Gallate(GCG)	2.290	-
Epigallocatechin (EGC)	3.076	-
Gallocatechin (GC)	5.132	-
Epicatechin (EC)	5.861	-
Epigallocatechin Gallate (EGCG)	31.429	-
Cellulose	46.60	100

The imaging study of fatty liver was done by experienced radiologist who was blinded to the all data of patients. The classification of NAFLD was based upon fatty liver grading on high resolution ultrasound machine (Toshiba Xario™ 200) ascited below.^14^

***Grade-0:*** Normal liver.

***Grade-1(Mild):*** There was normal diaphragm and intrahepatic vessel border with echogenicity of hepatic parenchyma was mild but diffusely increased or increased hepatorenal contrast.

***Grade-2(Moderate):*** There was slight impairment of diaphragm and intrahepatic vessel borders with moderate diffuse increase in the echogenicity of liver parenchyma and increasedhepatorenal contrast.

***Grade-3(Severe):*** Moreover to the above criteria. No visualization of diaphragm, intrahepatic vessel borders and posterior portion of the right lobe of liver.

### Data Analysis

A sample size (35 per group) was calculated to detect a difference of aminotransferaseslevel over 5 IU/Lwith 90% power and 5% significance. The sample size was increased to 40 per group to accommodate anticipated dropout rate. SPSS 16 was used to analyze data and their numeric values were expressed as mean ± standard deviation. A t-test was used to access difference between two groups at baseline. A paired t-test was used to compare the changes from baseline to 12 weeks with in each group while t-test or Mann-Whitney U-testwas used to compare changes between groups respectively. Values of p < 0.05were seemed to be statistically significant.

## RESULTS

The tolerability profile of GTE was good with no major adverse effects noted during the study period. However seven patients in GTE group had complaints of minor abdominal bloating during first week of therapy which were settled itself without any intervention andall patients completed the study. There was no significant differences in the baseline demographic features among two group at start of study Similarly there were no significant difference with respect to anthropometric parameters, lipid profile, aminotransferasesand fatty liver grading on ultrasound (Table-II). GTE resulted in a significant improvement in body weight, BMI, HOMA-IR, total cholesterol(TC), triglycerides (TG), low density lipoprotein cholesterol (LDL-C), high density lipoprotein cholesterol(HDL-C), alanine aminotransferases, aspartate aminotransferases, high sensitivity c-reactive protein (Hs-CRP) and adiponectin level after 12 weeks treatment as compared to placebo. These changes are shown in Table-III. Similarly GTE resulted in a significant improvement in fatty liver grading on abdominal ultrasound versus placebo. Although GTE improved all fatty liver grading but its impact was more on grade 1 and 2 fatty liver. The results are shown in Table-IV.

**Table-II:** Baseline parameters of Study Groups (N-80).

*Baseline parameters*	*GTE(n-40)*	*Placebo (n-40)*	*P-value*
Age(years)	25±18	28±15	0.71
Sex Male/Female	26/14	28/12	0.79
Body weight(kg)	85±12.5	79 ±14.5	0.68
BMI (Body Mass index kg/m^2^)	29.5±2.2	28.6 ±0.6	0.63
Systolic Blood pressure(mmhg)	110±5.4	115±5.4	0.05
Diastolic Blood pressure(mmhg)	78.5±6.2	76±8.2	0.03
Blood sugar fasting(mg/dl)	78 ±19.4	84±17.4	0.87
Fatty liver grading (1/2/3)	40(9/24/7)	40(8/27/5)	0.75

Values are given ± standard deviation, BMI: body mass index, LDL-Cholesterol, t-test: between two groups.

**Table-III:** Baseline parameters and outcome after treatment with GTE and Placebo.

*Parameters*	*GTE (n-40)*		P value*	*Placebo (n-40)*		P value*	*P value^+^*
	
	*Baseline*	*Outcome*		*Baseline*	*Outcome*		
Body weight(kg)	86±10.5	80±12.4	0.031	84±14.2	85±12.5	0.42	0.03
BMI(kg/m^2^)	29.5±3.8	27.2±3.2	0.004	29.5±1.5	30±0.9	0.82	0.026
HOMA-IR	4.32±2.25	3.16± 1.6	0.0056	4.56±2.61	4.82±2.33	0.26	0.0081
TC (mg/dl)	242.5±20.5	215.4±18.6	0.002	248± 22.4	252±25.4	0.77	0.005
TG(mg/dl)	175±22.6	145±18	0.006	187±20.4	189±22.5	0.96	0.003
LDL-C(mg/dl	155±12.5	140±16.7	0.001	160±10.6	162±12.7	0.55	0.011
HDL-C(mg/dl)	36.8±6.7	46.4±5.8	0.039	35.4±5.0	32±7.4	0.89	0.001
ALT(IU/L)	70.4±15.8	52.8±12.2	0.001	74±16.3	72±5.4	0.63	0.04
AST(IU/L)	65.8±12.4	44.3± 8.5	0.002	62.4±12.3	59±9.5	0.86	0.02
hs-CRP(mg/L)	3.14±0.58	2.18±0.32	0.009	3.16±0.58	3.21±0.76	0.93	0.023
Adiponectin(mg/L)	8.46±1.02	10.55±3.42	0.024	8.24±1.32	8.02±1.92	0.68	0.003

Results are expressed as mean ± standard deviation.

* P value indicate comparison within groups while

+ P value indicates comparison of changes of each variable between the two groups,

BMI: body mass index, HOMA-IR: homeostasis model assessment – insulin resistance index, TC: total cholesterol, TG: triglycerides, LDL-Cholesterol: low density lipoprotein cholesterol, HDL-cholesterol: high density lipoprotein cholesterol, ALT: alanine aminotransferases, AST: aspartate aminotransferases, hs-CRP: high sensitivity C-reactive protein.

**Table-IV:** Improvement in ultrasound finding of fatty liver after treatment with GTE and placebo(N=80).

*GTE group (n= 40)*			

*Fatty liver grading (ultrasound)*	*Baseline*	*Treatment*	*Outcome*
1	9	0	9
2	24	9	15
3	7	4	3

Total	40	13	27

***Placebo group (n= 40)***			

***Fatty livergrading (ultrasound)***	***Baseline***	***Treatment***	***Outcome***

1	8	4	4
2	27	22	5
4	5	4	1

Total	40	30	10

## DISCUSSION

In the present study, the effect of GTE supplementation on various parameters in NAFLD patients was estimated. To the best of our knowledge this was the first clinical trial performed on patients of NAFLD who were both non diabetic and dyslipidemic. There is no proven treatment for NAFLD patients currently available. There was significant improvement noted in BMI and body weight in GTE group at the end of study. Variousclinical studies have showed a positive impact ofweight reduction asan initial stepregarding NAFLD management.^6^,^15^,^16^ Green tea is very popularall over the world especially in Asia due to its weight reduction property. Obesity is the hallmark feature in most of the NAFLD patients and it is evident that even 5-10% weight loss is sufficient to decrease the amount of liver fat in NAFLDas well as to improveliver histology in NASH patients.^17^

The pathogenesis if NAFLD is poorly understood. Studies have shown that hepatic insulin resistancehas strong relationship with NAFLD. In order to overcome the insulin resistance, the two most studied drugs in NAFLD are metformin and pioglitazone and their efficacy was evident in meta analysisof numerous experimentaland clinicaltrials as both improves insulin sensitivity and reduces resistance in NAFLD.^18^ GTE also reduced insulin resistance by reducing HOMA-IR index and improved BMI and body weight in this study. The GTE also intended to decrease body weight by increasinginsulin sensitivity in obeseby activating AMPK pathwaylikemetformin^19^ and modulation of peroxisome proliferator activated receptors(PPAR) signaling pathwaylike pioglitazone.^20^ In addition GTE increases energy expenditure, increases fat oxidation, decreases nutrient absorption and decrease appetite that also has a strong influence on improving weight gain in obese patients.^21^

A meta-analytic assessment of NAFLD showed that about more than 50% of the patients are obese and dyslipidemic.^1^ In this study there was significant improvement in lipid profile in correlation with BMI and body weight. The proposed mechanism by which GTE produces beneficialeffects on serum lipid profile is due to its EpigallocatechinGallate(EGCG) componentwhichforms complexes with lipids and lipolytic enzymes, thereby interfering the process of luminal emulsification, hydrolysis, micellarsolubilization and subsequent uptake of lipids.^22^ Research also suggests linalool and EGCG improves lipid profile and obesity by acting on peroxisome proliferator activated receptor (PPAR-α) which is the site of action of standard lipid lowering agent gemfibrozil.^23^

The influence of anti-inflammatory and anti-oxidant agents such as vitamin E and silymarinfor improvement in serum transaminases in NAFLD patient’s givesclue about the possible involvement of inflammation and oxidative stress in the pathogenesis of NAFLD.^7^ In this study GTE caused a significant reduction in hs-CRP level. The reduction of inflammatory marker by the GTE causes the significant improvement in liver enzymes. Similarly GTE also significantly increased level of adiponectin in this study. Adiponectin is a hormone whose biosynthesis is deranged in obesity, metabolic syndrome, Type-2 diabetes, inflammation and NAFLD. Adiponectin also possessespowerfulanti inflammatory andanti oxidant properties as it may antagonize the effect of TNF-α and attenuates the progression of NAFLD by decreasing the proliferation of hepatic satellite cells and increases apoptosis.^24^

Most of the studies of GTE on NAFLD were experimental in which GTE improved body weight, lipid profile, blood pressure, aminotransferases level, inflammation, oxidative status through multiple mechanisms. 25 Thereare limited studiesconducted on green tea in patients of NAFLD. A study conducted by Pazeshkiet al.^26^ showed that GTE supplementation decreases aminotransferases level in patients with NAFLD for a period of 12 weeks. Our results were almost similar to studies conducted by Sakata et al.^27^ and Fukuzawa et al.^28^ which showed that GTE not only decreased liver enzyme level but also improved body mass index (BMI), blood sugar, lipid profile, body fat percentage, radiological findings and markers of oxidative stress and inflammation in patients with NAFLD for 12 weeks and NASH for 24 weeks. Moreover this studyincluded overweight, non-diabetic patients with insulin resistance and dyslipidemia, which also showed that GTE has also independent impact on NAFLD irrespective of its glycemic control effect. So GTE can be useas dietary supplement to prevent and control NAFLD in diabetics as well as in non diabetics.

## CONCLUSION

GTE capsule 500mg twice daily improved NAFLD associated changes in non-diabeticdyslipidemic patients.

## RECOMMENDATION

GTE is a good dietary therapeutic option in NAFLD patients. Further clinical trials of long duration should be conducted to confirm our observations.
